# Hammond on Chancre

**Published:** 1861-10

**Authors:** 


### HAMMOND ON CHANCRE.

*From Cleveland Med. Gazette and American Med. Times*

From several lectures delivered at the Baltimore Infirmary, by Prof. Wm. A. Hammond, we condense the following statements in regard to the nature and treatment of chancre:

There are two species of venereal poison: one giving rise to a simply, non-infecting, soft chancre; the other causing an indurated one, liable to be followed by constitutional syphilis. There are also two kinds of virulent gonorrhœa, corresponding to the kind of venereal poison which comes into contact with the mucous surface of the urinary passages. The two poisons are by no means convertible into each other, but are essentially distinct, each kind of chancre inoculating with its own specific poison, and causing a sore of the same character as the parent chancre.

The soft chancre has never an indurated base; has perpendicular edges, a rough, dirty gray surface; discharges pus generally of a healthy character; enlarges to the size of a dime, then cicatrizes and heals in about four weeks. It is a local disease, never infecting the general system; the secretion from it may be inoculated, as long as the process of reparation has not advanced far. But this chancre is pre-eminently liable to complications: inflammation from irritating substances improperly applied (corrosive acids, nitrate of silver, sulphate of copper, etc.), or from individual causes, such as debility, dram-drinking, or the inordinate use of mercury, or from accumulation of the discharge behind the prepuce, or mechanical irritation, friction against the clothing, or during coition. Ulceration may occur, even without increased inflammatory action. Phagedena is due to constitutional causes, intemperate habits, excessive sexual indulgence, bad food and air, but above all, to the influence of mercury. The bubo occasionally accompanying soft chancre is either a simple symptomatic adenitis, and non-virulent, or caused by the absorption of pus, then always of a specific character, suppurating, and furnishing an inoculable secretion.

It is always desirable to destroy the specificity of the chancre at an early stage. Of all the caustics recommended for that purpose, none is so manageable, and at the same time so effective, as the paste of sulphuric acid and charcoal, recently recommended by Ricord. Take strong sulphuric acid and mix finely powdered charcoal with it, so as to obtain a paste, which is carefully applied over and around the chancre.

In three or four days it falls off, with the slough it has produced, leaving underneath a healthy sore. The pain caused by the application, may be mitigated by opium. Where the paste cannot be employed, as in the uretha or rectum, nitric acid should be used, and a dossil of lint, thoroughly soaked in olive oil, be inserted afterward. A soft chancre in the process of healing, requires astringent or slightly stimulating applications, one of the best of which is a weak solution of tannin in water. Sulphate of zinc, acetate of lead, nitrate of silver, may also be employed, in weak solution, keeping the sore constantly moist with them. A constitutional treatment is rarely required. The bowels should be kept open, and indigestible food avoided, but no other special precautions are necessary. Wine, coffee, beef-steaks, cigars, etc., ought not to be forbidden to patients in the habit of using them. The most perfect cleanliness is desirable.

When inflammation is present, emollient applications, such as mucilage of flax seed, warm water, poultices, etc., are to be used. One of the best and most elegant articles is a cataplasm of chamomile flowers, changed frequently. Perfect rest in bed, a mild purgative, and a grain of opium every four or five hours, complete the treatment. The tincture of the chloride of iron, in doses of from thirty to forty drops, four or five times a day, is often of decided benefit, especially when there is a tendency to gangrene. The abstraction of blood is hardly ever required, while stimulants are generally of good service.

When phymosis or paraphymosis results from the inflammatory engorgement, warm applications and a soothing treatment do all that is necessary. Should, with phymosis, gangrene threaten, run a director under the prepuce and slit it open with the probe-pointed bistoury, till the constriction is removed. In paraphymosis, if mild attempts at reduction fail, and mortification threaten, the stricture should be divided by running a straight, sharp-pointed bistoury under it, and cutting outward.

For excessive ulceration, the sulphuric acid paste is generally the best application, with a grain of opium night and morning, internally. If the sore produced by the escharotic shows a tendency to spread, strap it with adhesive plaster, and give a chalybeate with opium.

The serpiginous ulcer is difficult to cure. Fowler's solution internally, and arsenious acid, with sugar sprinkled over the sore daily, frequently cause a very favorable change. Iodine, however, gives more satisfaction. Give Lugol's solution internally, with some preparation of iron, or with cod-liver oil, and apply the tincture to the ulcer and neighboring parts every day. With this treatment the most obstinate serpiginous ulcers rarely fail to improve and heal.

Against phagedenic chancre, the sulphuric acid paste is the best remedy, with good diet, plenty of fresh air, and some preparation of iron. The potassio-tartrate seems to be almost a specific. Dissolve one ounce of it in ten ounces of water, and give of this, half an ounce three times per day, keeping at the same time, the diseased part constantly moistened by it. Some cases not early attended to, resist all treatment.

Bubo from simple adenitis, advances slowly, is not painful, and soft. Should it break, or be opened, the pus is discharged without inoculating the edges of the wound. The virulent bubo is of rapid growth, suppurates early, and is accompanied with considerable pain; the integument covering the abscess, if not punctured, sloughs, leaving a true chancre, with inocuable pus. This chancre in the groin is more liable to phagedena than the original sore. The simple form is to be treated with discutient lotions, containing sub-acetate of lead or chloride of ammonia. These may be conjoined with pressure by means of a graduated compress, held in its place by a proper bandage, or by collodion painted over. The tincture of iodine, or the ointment, are also valuable applications; still better is a solution of iodide of potassium, twenty grains, and iodine forty grains, in one ounce of glycerine; or the iodide of lead ointment may be used. If dissolution is not effected by these means, an early incision should be made, or rather, several small setons be passed through the back of the swelling, followed by pressure. Pyæmia may result from too large an incision; a tedious recovery certainly does.

In the virulent bubo, suppuration must be hastened by chamomile cataplasms, or other poultices, and as soon as possible the abscess should be laid open by a long incision. The remaining ulcer is treated with the escharotic paste, etc., in the same manner as the original chancre.

The indurated, infecting, or true syphilitic chancre, appears but once in a lifetime, on the same individual, and the secretion from it cannot be successfully inoculated in the body of the patient. When this form has commenced to heal, it, like the first, loses its virulent characteristics entirely. An indurated bubo is an almost constant companion, but may, in some cases, be prevented by a timely administration of mercury. Secondary symptoms may ensue without the presence of a bubo. A characteristic sign of this kind of bubo is the enlargement of all the lymphatic glands around the infected one.

The principal indication of the treatment, is to prevent infection, the cure of the local trouble being comparatively a small matter. If seen in the first stages, say within the first six days from the appearance of the pustule, the indurated chancre should be destroyed by the sulphuric acid paste. After cauterization, the most simple applications are all that is necessary. The aromatic wine acts very well; a weak solution of tannin is equally efficacious. Should the chancre be healing, or have ceased to progress, cauterization is useless and improper. In such cases, commence the local treatment with the solution of tannin. Nothing else is required locally. The efficacy of internal remedies in accelerating the cure of indurated chancre is doubtful, although they may be necessary for the purpose of preventing infection, or destroying the morbid matter circulating in the blood. Indurated chancres heal just as soon without mercury as with it. Under the influence of this agent, the induration disappears sooner than if no mercury is given, but the ulcer is just as long in healing.

